# Towards the contributing factors for stress confronting Chinese PhD students

**DOI:** 10.1080/17482631.2019.1598722

**Published:** 2019-04-25

**Authors:** Xueyu Wang, Chun Wang, Jian Wang

**Affiliations:** a School of Foreign Languages, Huaqiao University, Quanzhou, the People's Republic of China; b School of Civil and Commercial Law, Southwest University of Political Science and Law, Chongqing, the People's Republic of China; c School of Foreign Languages, Southwest University of Political Science and Law, Chongqing, the People's Republic of China

**Keywords:** Grounded theory, Chinese PhD students, stress, contributing factors, suggestions

## Abstract

**Purpose**: A review of literature reveals that stress is prevalent among PhD students who are experiencing higher levels of stress than age-matched general population normative data, and has drawn attention worldwide. However, few studies have examined the factors influencing the psychological well-being of Chinese PhD students and the type of supports most needed.

**Method**: This study was conducted by a qualitative method employing the Grounded Theory. Through purposive sampling, 10 Chinese PhD students were selected on the basis of theoretical sampling, and data were collected through semi-structured, in-depth interviews with the participants.

**Results**: Chinese PhD students experienced stress of nuanced nature at their varied years of PhD study. The obtained codes were categorized under four themes, including graduation, job prospects, relationship and other factors.

**Conclusion**: Chinese PhD students experienced stress from a variety of sources, corroborating with and reinforcing previous research findings. By exposing the explanations of the factors for stress confronting Chinese PhD students, this study compels us rethink the relationship between the widely existing stress and relevant policy or regulations, and proposes suggestions for counselling and policy reform.

## Introduction

A PhD, as the highest degree in academic career one could have, is a title that carries prestige and respect; as a result, PhD holders, who are the backbone of the research industry, have earned the societal right to be addressed as “Doctor”; as a result, they can make more money on average compared to those without, and are always admired and envied by those who do not have a PhD. With so many encouraging stories around, it is easy to overlook stress that lies hidden behind a superficial glory of having a doctoral degree. The astonishing fact is that PhD students, faced with a large number of challenges during their candidature, have to forgo much toil, many tears, as well as sweat even blood to jump through many hoops of graduate school!

Compared with other professions or occupations, sources of stress, anxiety, apprehension or exhaustion (hereinafter collectively referred to as “stress”) in academia are more prominent, as is evidenced by the findings of two studies, conducted in 1998 and 2004, of academic staff in British universities, which revealed that academics were often more stressed than nurses and social workers and there had been little change in the levels of most stressors experienced over the 6-year period (Kinman, Jones, & Kinman, 2006).

Further, numerous researches or reports in journals or social networking websites also unveil the hardship and toil experienced by PhD students during their candidature. A recent survey of a sample of 3659 PhD students at Flanders (Belgium) reveals that students face significant mental health challenges. According to the survey, approximately one-third of PhD students studying science and social sciences are at risk of having or developing a common psychiatric disorder like feeling under constant strain, being unhappy or depressed; 51% of respondents had experienced at least two symptoms of poor mental health in recent weeks leading up to the study, indicating psychological stress. Moreover, 32% reported at least four symptoms, indicating a risk for procuring a common psychiatric disorder, which was more than twice the prevalence among highly educated comparison groups (Levecque, Anseel, De Beuckelaer, Van der Heyden, & Gisle, 2017; Pain, 2017). Further, using validated survey instruments, Barry, Woods, Warnecke, Stirling, and Martin (2018) found that PhD students reported higher levels of depression, anxiety and stress than age-matched general population normative data. These apart, other researchers vividly described the stress or anxiety experienced by PhD students and the causes for stress in different countries, incorporating qualitative and quantitative components into research (e.g., Appel & Dahlgren, 2003; Bazrafkan, Shokrpour, Yousefi, & Yamani, 2016; Bi, 2007; Castle, 2013; Haksever & Manisali, 2000; Jackson, 2013; Kurtz-Costes, Helmke, & Ülkü-Steiner, 2006; Levecque et al., 2017; Lu, Wang, & Tian, 2012; Ülkü-Steiner, Kurtz-Costes, & Kinlaw, 2000; van der Haert, Ortiz, Emplit, Halloin, & Dehon, 2014; Wisker & Robinson, 2013).

Chinese PhD students are no exception in this respect. On March 11 of 1998, a female Peking University student suffered psychological stress and committed suicide (Feng, 2018); On 25 December 2017, a second-year male PhD candidate studying pharmacology at a prominent university in north-western China also committed suicide, allegedly due to enormous pressure he faced at the university (Wang, 2018). These cases, along with other reports on social media, though just the tip of the iceberg, have once and again highlighted concerns for psychological stress experienced by Chinese PhD students, demanding more immediate attention from pertinent authorities.

Anyway, the available studies reveal that psychological stress is highly prevalent in PhD students who are experiencing various stress or anxiety or other psychological health problems (e.g., suicide attempts, acute situational stress reactions, or insomnia) during their candidature, and, exerts negative impacts on PhD students. Nevertheless, psychological stress among PhD students in institutions of higher education is a relatively unexplored area of research in China, especially lack of research under grounded theory. In this study, a grounded theory approach is used to delineate and understand the sources of, or causes for, stress among PhD students, so as to inform the type of supports most needed to address the issue.

In this study, stress is confined to negative emotional experiences that can make people feel exhausted and edgy, steal their focus, or leave them spending their nights sleepless and frantic, not including distress or psychological disorder in psycho-oncology literature as suggested by Stein et al. (2010).

## Methods

### Research design

This study is not intended to measure the ratio of stress experienced by PhD students and provide further rigorous statistical data for future study, so a qualitative approach using the grounded theory proposed by Strauss and Corbin (1990) is adopted, to provide a general framework to explain why Chinese PhD students are experiencing stress.

To streamline and validate semistructured one-on-one interviews that were to be used in the major study, a pilot study was conducted due to its various functions (Dikko, 2016).

First, we determined clearly initial interview questions on issues related to causes for psychological stress. The interview protocol, encompassing semi-structured and open-ended questions, was sent for expert reviews of its wording and relevance, and was modified accordingly. Then, interview questions were tested in the pilot work, and we used probing questions to get further views or clarifications from the participants;

Second, we carefully selected participants who were introduced to us by our colleagues, friends and classmates and conducted piloting interview. To ensure the informants to share as similar criteria as possible to the group of participants for the major study as suggested by Hennink, Hutter, and Bailey (2011), we had given the inclusion criteria of participant beforehand. So, the informants were selected based on purposive sampling and willingness to participate. An effort was made to interview two PhD students, one male and one female, in the first and third year of study respectively. The pilot study to explore their experiences was conducted in early 2017. The interviews were conducted in a quiet café near their respective universities, and commenced with social conversations, in the presence of the friends who introduced the informants to us so as to build rapport with the informants. The interviews were recorded using smartphones, ranged in time between approximately 30 min and 1 h. Of course, informed consent or approval was obtained from both informants.

The pilot test offered us an opportunity to refine and clarify some interview questions. For example, “why” questions were replaced with “how” questions (How did you come to do a PhD?) to get stories of process rather than acceptable accounts of behaviour, and the logical flow of the interview was reconsidered (instead of asking question in the protocol in a sequential way); Further, pilot test also helped draw our attention to questions ought to have been included in the interview guide interviews (e.g., embarrassing questions were added towards the end of the interview, when rapport had been established). Anyway, the pilot study pretested the appropriateness of the interview questions, provided us with certain early suggestions on the viability of the research, helped us obtain experience in conducting in-depth, and learn the skills in interviewing and the flow of conversation.

### Participants

Following the pilot test which determined that the interview protocol satisfied the requirements for validity and reliability within the parameters of a qualitative research, the major study commenced. In-depth, semi-structured interviews were conducted with 10 participants recruited through seven universities in three provinces of the People’s Republic of China. Purposeful sampling of interviewees occurred chosen at the outset of the study, subsequently, theoretical sampling was adopted until theoretical saturation of the main body of data occurred (Charmaz, 2014). The Demographic characteristics of the participants are depicted in Table I.

**Table I.** Demographic characteristics of participants.

Participant’s code	Age	Gender	Period of PhD training	Relationship status
No. 1	26	Male	1st Year PhD Student	Single
No. 2	27	Female	1^st^ Year PhD Student	Single
No. 3	28	Male	2^nd^ Year PhD Student	Married
No. 4	32	Female	2^nd^ Year PhD Student	Single
No. 5	29	Female	3^rd^ Year PhD Student	Single
No. 6	42	Male	3^rd^ Year PhD Student	Married
No. 7	48	Male	4^th^ Year PhD Student	Married
No. 8	36	Female	4^th^ Year PhD Student	Married
No. 9	40	Female	5^th^ Year PhD Student	Married
No. 10	39	Male	5^th^ Year PhD Student	Married

### Ethical considerations

In China, there is no IRB (Institutional Review Board) in the American sense, but at the outset of the interview, each participant was informed of the purpose of the study, and that the interview would be taped and take approximately half to 1 h. Further, they were informed of the voluntary and confidential nature of the interview, i.e., their name, affiliation, residence or other personal information would be completely anonymized in the research, and that they may refuse to answer questions or drop the interview at any time.

### Data collection and analysis

We interviewed the participants individually in private places at different time in the past 12 months, by asking them open-ended questions in a face-to-face manner. These semi-structured, in-depth interviews varied in length from 30 min to nearly 1 h and were audio recorded, then each interview was allocated a number to its transcript.

The interviews were conducted in Chinese and translated in English, then back-translation method was used to ensure linguistic equivalency of instruments before and after translation (Brislin, Lonner, & Thorndike, 1973). The interview transcripts were compared with each other for common words, phrases, and concepts through constant comparative method. Under this constant and dynamic process, newly collected data were compared with previous data collected by other scholars in earlier studies, and formed, enhanced, confirmed or even discounted theories.

Two strategies proposed by Creswell (2007) were employed to establish credibility of the data: (1) member checking, i.e., the participants were provided with the data or interpretations of the results to verify the accuracy based on their experiences; and (2) peer review, i.e., two external qualified researchers were requested to examine the research processes and data interpretations and verify that the data were collected and analyzed in an appropriate manner.

The process of analyzing data included coding at three levels (Strauss & Corbin, 1990). After intensive collection of data, we began to identify, name, categorize and describe phenomena found in the texts. We analyzed interview transcripts to develop a set of “open” codes that marked the key points revealed in the interviews. Sarker, Lau, and Sahay (2000) remarks, the emerging large number of categories/sub-categories inevitably entails difficulty in distinguishing between properties and sub-categories in many instances. To avoid this problem, we reviewed and compared these codes in a group, and clustered them into conceptual categories. After reviewing and revising the codes and categories, we embarked on a process to test the codes for clarity and reliability. A sample set of transcripts was submitted to a team which was made up of a professor who had been publishing articles in this field regularly and recently, and two graduate assistants. We met them once a week for 4 weeks to discuss coding of data, applying the constant comparison approach. In this way, the commonalities or distinctions among the meanings of similar data became clear, and we rearranged the properties of each code, condensing some codes into one, or developing new codes. Based on intensive open coding, selective coding was used to arrange key phenomena which emerged from the data into grouping (Strauss & Corbin, 1990) to develop the categories. Further, story lines were conceptualized to identify the categories and make links between the categories, as is depicted in Table II.

**Table II.** Final Coding System.

Category	Subcategory	Codes
Graduation	article publication	supervisor’s omission; publication policy; excess demands; personal reasons
	thesis writing	time limit; course loads; supervisor’s omission; thesis proposal; oral defence; spot-checking; personal reasons; etc.
Job prospects	job expectations	“Program 211 or 985” universities; good-salaried jobs;
	employment situation	enrolment policy; employment situation; sparse job vacancies; job expectation failure; PhD surplus; fierce competition; graduates of Program 211 or 985” universities; PhD returnees; gender discrimination etc.
Relationship	supervisor–student relationship	conflicts due to relationship of professional nature or personal nature
	family or marriage	familial relationship; fear of being left on the shelf; prospective spouse
	relationship with roommates	conflicts due to different living habits, envy, etc.
Other factors	personal factors	personal character; sex; outsider’s expectations, own expectations, perceived academic status, etc.
	financial factors	poverty; limited PhD program funding; wealth of non-student peers

## Results

Although the participants varied in a number of social or cultural dimensions (e.g., family social class, relationship status, gender, etc.), but their stories overlapped or converged. They all acknowledged that the extent or degree of stress experience was different for each individual but shared some common characteristics. For example, Participant No.10 remarked, “No people may be immune to stress. Once you choose to do a PhD, you will experience stress or anxiety of this kind or that… and that’s for sure.” During the interviews, participants dictated varied source of, and factors for stress. For example, Participant No.9 said, “One of my classmates is crazy about article publication… he can’t submit his dissertation proposal for lack of articles; another classmate has trouble concentrating on dissertation writing…as a leader in his *danwei* (单位, meaning an institution where one works), he has got lots of administrative work to do…, while what stresses me out most is I must publish one more paper in the coming year, for this is the fifth year of my PhD program. Otherwise I would be terminated from the doctoral program”. After all, according to the *Procedures for the Award of the Doctor of Philosophy Degree* promulgated by most universities in China, all program and university requirements for the PhD must be completed in 6 years from start of the doctoral program. Failure to meet the requirements for the PhD within the timeframe delineated is interpreted as a withdrawal from the doctoral program or failure to graduate, and no doctoral degree will be conferred.

**Figure 1.**
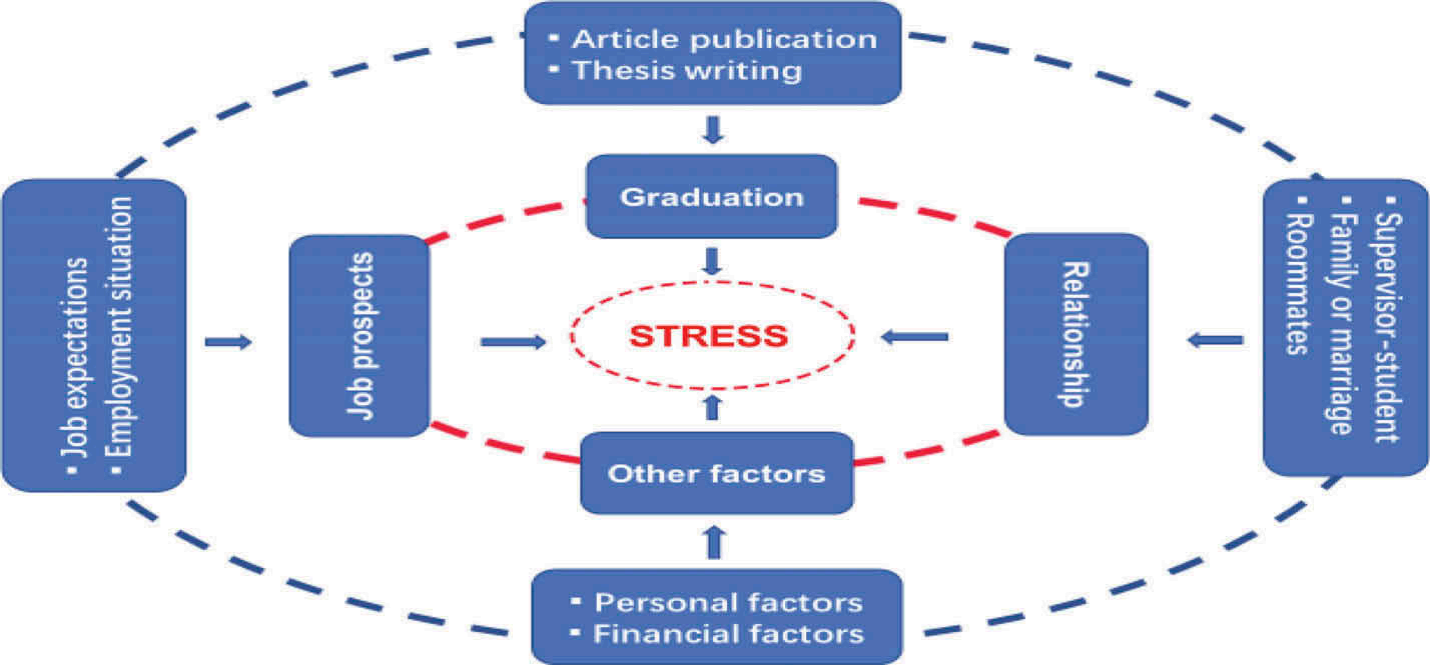
Contributing factors for stress.

The GT emerging from the data is considered to be at substantive level as suggested by Glaser and Strauss (1967), for the explanations we have derived for psychological stress apply specifically to Chinese PhD students (majoring in social science) in the setting that we studied. The theory depicted vividly the nuanced nature of participants’ experiences of stress. Although the participants’ individual circumstances varied to a certain degree, all of them deemed psychological stress as an indispensable experience which accompanies their lives as PhD students. Their experiences of psychological stress were complex, and they discerned that stress mattered a lot to their lives as PhD students. To some participants, one single factor could account for their stress; but to most participants, their problem should be understood as reflecting a complex set of triggers that combine aspects of more than two explanations. And the more stressors they experienced, the more stressed they tended to feel, i.e., the interactions among these factors severely increased their degree of stress. The results revealed that the concepts of causes for psychological stress converged into four core categories, i.e., graduation, job prospects, relationship and other factors, and each category contained its subcategories and properties, making contributing triggers for psychological stress, separately or collectively (see Figure 1).

### Graduation

In China, full-time doctorate program is normally designed to be completed in 3 years, but the program duration of a professional doctorate is rather flexible, i.e., comprising a minimum of 3 years full-time-equivalent (FTE) study and a maximum of 6 years from the date of commencement of candidature, so PhD students have adequate time to complete the work for the degree and submit the thesis, totally in command of every situation. However, the government scholarships (covering the intuition fee) and incentives (aimed at helping support the living costs of PhD students) for PhD programs usually last at most 3 years. Namely, if students fail to graduate within 3 years, they no longer receive any funds to cover any expenditure incurred during candidature; consequently, they must do something to sustain themselves financially or to make ends meet, which in turn takes up much time they should spend studying. As a result, all PhD students are eager to graduate on time, for graduation within the time limit not only means a saving of time and money, but also is an indicator of personal competence; after all, in people’s eyes, only the most outstanding or distinguished students can graduate on time.

During the interviews, participants repeatedly asserted that pressure to graduate on time was the key cause for stress. Each participant was worried that failure of degree conferral within the 3-year time limit would entail termination of his/her PhD program. Completing a PhD is hard, and getting harder, as universities attach greater importance to the domestic ranking which is mostly based on research quality and revenue (e.g., the number of projects at national level and publications in core periodicals). In accordance with the procedures and criteria for the conferral of a doctoral degree widely applicable in almost all institutions of higher education in China, doctoral degree is conferred on two conditions, i.e., (1) a thesis of no less than 100,000 words, which should be independently developed and publicly defended, containing original research results and new insights in their respective fields or subfields; and (2) at least two articles published or accepted for publication in core periodicals in the fields related to their major (i.e., core periodicals listed in Chinese Social Science Citation Index, hereinafter “CSSCI”) or periodicals listed in SSCI.

Near all the participants asserted that it was hard to satisfy the first requirement, due to personal reasons (e.g., not intelligent enough to complete a good-quality thesis), supervisor omission, or other provisions. During the 3-year doctoral program, the first year started with heavy duty coursework and a lot of specific requirements, such as attending training seminar led by the supervisor, participating in research projects conducted by the supervisor, participating various social activities, etc., and ended with qualifying exams on all the core tutorials or lectures. In the second year, students can shift to conducting independent research by submitting thesis proposal. Almost all students agreed that they were suffering or had suffered the common ailments known as PhD fatigue and PhD blues when choosing a subject satisfaction to themselves and their supervisors, as Participant No. 3 put it, “…my supervisor wanted me to choose a subject which could sustain interest for several years…I have to spend days alone in front of my computer, surfing the internet. Now, 5 months have passed, the topic is not as gripping as initially believed. I am desperate, not knowing what to do.” Participant No. 4 also remarked, “… thesis proposal rarely passes without an objection from your supervisor requiring a modification to the proposal. I have modified my thesis proposal for 3 times before attending thesis proposal defence. It really drove me crazy.” Students would be in possibly a more desperate situation when their supervisors gave no or little advice for revision and merely refused to let them stand for a defence. This is the case with Participant No.8, who submitted her thesis proposal for four times, only to be rejected by her supervisor who, obsessed with his career as lawyer, gave no suggestions for revision. Feeling extremely exhausted and suffering from severe insomnia, she was considering withdrawing from the PhD program.

Stress also came from vigorous blind peer review by external examiners selected by the graduate school board, failing which graduation would be postponed, and originated from the oral defence when students were at the end of a long period of grappling with dozens of tricky and detailed problems and issues pointed out by internal examiners. Just as Participant No.5 said, “I was so anxious about the defense, for some weeks before the oral defense, I thought ahead to the issues that examiners would have, for example, why you chose to adopt this approach instead of that. These kinds of concerns buzzed around my head, even prompted me to lie awake at night rehearsing answers to the examiners.” Further, stress also originated from the spot-checking arranged for by the Ministry of Education, failing which the already awarded degree would be cancelled.

Meanwhile, no participants denied that it was even harder to satisfy the second requirement. To publish two articles in non-core periodicals were nothing hard, but to get two articles published in periodicals listed in CSSCI or SSCI was another thing, even impossible when they received no help from supervisors. At a time when publication output has become a milestone for academic selection and promotion (e.g., conferring of all academic titles), access to future research funding, or professional development opportunities in institutions of higher education in China, it is really a great challenge or unfair for PhD students to compete with numerous academic scholars to get articles published in limited core periodicals. As a result, students were desperate for publication. Just as Participant No. 6 said, “…My manuscripts have been rejected by five periodicals, while my supervisor does nothing about it. I’d love to pay RMB 30,000, or even more, to a periodical listed in CSSCI in exchange for publication of one article, if it is possible.” When talking of article publication, we obviously perceived a pervading mood of gloom and sullenness among the participants.

### Job prospects

Job-related stress mainly lied in the discrepancy between the career aspirations and the actual situations of employment. Interviews revealed that students had a varying spectrum of motivation for choosing PhD program, e.g., to find a job in outstanding institutions of higher education, research institutes or prominent institutions or corporations, get promotion, receive a pay raise, etc., consequently to change their social status and improve their living standard. It was and still is widely perceived among the PhD students that a PhD degree means higher career aspirations and expectations. After all, the PhD forms a watershed period where PhD candidates’ professional identities are established and their career aspirations and expectations are developed (Dufty-Jones, 2018). But students were baffled or unhappy with their career opportunities, for they pursued PhD study to secure a proper job to their heart’s content (e.g., work in leading universities; settle down in such metropolitan cities as Beijing, Shanghai, Guangzhou, etc.; find a high-paying job), only to find that even with a PhD, their hopes of finding a good job were as dead as a doornail. In a sense, to pursue a PhD itself is an indicator that PhD students fear the difficulty of finding proper jobs, so a safe conclusion can be drawn that this stress starts even before their PhD studies! Interview revealed that job-related stress was attributable to the following reasons.

Firstly, surplus of doctorates in academia made good job shadowy. Most PhD students aspired to work in academia, especially in prominent institutions of higher education or research institutes like “Project 211” Universities consisting of about 100 institutions of higher education and key disciplinary areas or “Project 985” Universities comprising 39 world-class universities in China (hereinafter “Project 211 or 985” Universities, which functions as government initiatives to categorize the top universities in China), but they were pessimistic about their prospects of doing so, due to the ever-outstanding surplus of doctorates compared to traditional academic jobs in China. According to the statistics revealed by the Ministry of Education of the People’s Republic of China, 53,139 people graduated with a PhD in China in 2014, compared with 53,653 in 2015, 53,778 in 2016, and 55,011 in 2017 (see: en.moe.gov.cn). Meanwhile, in 2014, 2015 and 2016, the numbers of teaching staff in all institutions of higher education or research institutes increased by 37,600, 38,100 and 29,400 (including those graduates with master’s degree), respectively (see: en.moe.gov.cn), which means more than 30% of doctoral graduates each year failed to work in academia as they expected;

Second, the fear of lack of personal competitive force intensified their stress or anxiety. PhD students of non-“Project 211 or 985” Universities, compared with those of “Project 211 or 985” University, had greater stress or anxiety, due to the fact that the latter were recognized as superior to the former, for “‘Project 211 or 985ʹ University graduates only or preferred” was highlighted by staff recruitment advertising on the websites of almost all institutions of higher education; Meanwhile, PhD students of “Project 211 or 985” Universities felt stressful when competing with PhD returnees. Since the early 1990s, Chinese government has given generous funds to Chinese “star” academics working abroad if they return to China; In 2005, China established “Plan 111”, which was set out jointly by Ministry of Education and State Administration of Foreign Experts Affairs, intended to bring in about 1,000 overseas talents from the top 100 universities and research institutes worldwide and to upgrade the scientific renewal and peers’ competition of Chinese universities by establishing innovation centers and gathering groups of first-class minds from around the world. According to the Report on Employment &Entrepreneurship of Chinese Returnees 2017 produced by CCG (Center for China and Globalization), the number of Chinese returnees registered at Chinese Service Center for Scholarly Exchange reached 370,000 in 2014, of which 22,000 held a PhD, and the number of returnees reached 432,500 in 2016, and would reach 666,000 in 2017, as predicted by the Ministry of Education (see: http://www.xinhuanet.com/fortune/2015-06/29/c_127961858.htm). In recent years, higher education landscape has become even more increasingly competitive, as colleges and universities strive to recruit and retain high-quality graduates to be qualified as “Double First-Class University” (Chinese:双一流大学), which is a plan conceived in 2015 to create world-class universities and disciplines in China by the end of 2050. Thus, those foreign graduates and Chinese returnees holding a PhD from western top universities and several academic publications get the edge on domestic PhD graduates, for they can be readily recruited by top universities in China and appointed at a higher level, or even at full professor level immediately.

Further, female PhD students were more stressed than their male counterparts, for fear of gender discrimination in job seeking. According to the All-China Women’s Federation which has been monitoring the discrimination issue in employment, gender discrimination has become acute as a record number of graduates are chasing work in a tight job market (Chen, 2017; Sharma, 2013). Anecdotal evidence, like the *Cao Ju* case involving gender discrimination in universities and the workplace, suggesting that female graduates are having a more difficult time (Branigan, 2014), certainly casted shadow on female PhD students.

### Relationship

This category was further divided into 3 subcategories. The first was the strained supervisor-student relationship. Some supervisors sometimes were academically enabling and supportive, for example, helping set out schedules for paper or thesis writing, but the interviews revealed that more often, supervisors were also bullies. Some of them did not meet students regularly (e.g., Some PhD students said they never saw their supervisors at all, so they just got on with the work themselves), but made unrealistic demand on their progress (e.g., asked students to finish reading a lot of books within a semester and write study reports); alternatively, some supervisors showed little interest in students’ work, provided no feedback to students’ academic inquiry, repeatedly disapproved thesis proposal without any suggestions, committed sexual harassment ranging from verbal abuse to molestation, required students to help do research work or teach in supervisor’s stead, or even required students to do some personal things irrelevant to study (e.g., asked students to pick up supervisor’s kids from school, asked students to apply on supervisors’ behalf to the financial department for the reimbursement of all personal or travel expenses and other conference costs, kept pushing students to drink late at night or during the day, etc.). Some participants exclaimed that this tricky relationship that fell into either of the cases above was a contributory factor in increasing their stress.

Second, family or marriage concern, including mate selection criteria, age for marriage and child-bearing age, and relation between family members, also triggers stress. Most single PhD students remarked that theoretically speaking, a higher academic degree should have enabled them to be more advantageous in mate selections (e.g., age or academic degree). But ironically, they could not take out time from their busy schedule to date frequently. As they were growing old, mate selection range would dramatically narrow down, consequently, they felt greatly stressed. Compared with male students, female students, frightened of being left on the shelf, were all the more stressed out. As Participant No. 4 said, “…So busy with my PhD study, I haven’t got a boyfriend yet. I am over 30…I don’t know when I can tie the knot and give birth to a child. … I like child, but I am worried that no one would marry an old maid.” This worry was quite prevalent among female single PhD students who were struggling between career, study and marriage, and worried that while they might succeed in study, they would lose their lifelong happiness due to the contraction of prospective better halves. After all, in Chinese culture, it is quite shameful for a man to marry a woman who is superior to him, especially in respect to academic degree; Meanwhile, married participants were troubled by complaints or dissatisfaction from their family members, for throwing themselves into PhD study thus not accompanying or caring for the family, having nearly no social life, spending so much money on buying books, etc. Some family members even suspected it was worthwhile to embark on a PhD at a sacrifice of time, energy, money, happiness, etc., which inevitably resulted in fighting and bickering between PhD students and their family or mates. Some participants remarked that it was stressful to strike a balance between family and study. Their role as a parent, spouse or mate was considered to be a major stumbling block to realizing their PhD study. As Participant No. 7 said, “Being a father and husband while trying to complete a PhD is really tricky. I must devote more attention to my study to graduate on time. That is the top priority. But my wife always complains for not caring about her and our kids …she doesn’t know I am in a terrible situation. People who haven’t done PhDs cannot understand why we still want to read theses or articles at night—when we had the whole morning and afternoon to do it. Her lack of understanding drives me crazy!”

The third factor is the tension between roommates. Data revealed that tension between PhD students was unavoidable when they shared a dorm, due to different personal hygiene habits, living habits or personal characters, e.g., staying up late, being hospitable (so receiving friends and dining in the dorm frequently), snoring loudly, smoking in the room, etc. As a result, participants involved felt stressed out, and could not focus on writing or getting enough sleep, and even suffered from insomnia when mediation failed and the relationship between roommates became more intense.

### Other factors

During the process of axial coding, we found some sub-categories overlapping and converging. Some factors were not uniformly applicable to all the participants, namely, only part of the participants claimed these factors to be the sources of stress. Following the suggestions of Strauss and Corbin in case of tight integration and dense development between core categories and subcategories, “financial factors” was categorized as “other factors”.

The above-mentioned factors apart, there were other contributing factors for their stress. Three participants remarked that they were in a stressful plight whether to continue or quit. Just as Participant No.2 said, “I am not well-prepared for the PhD study, not recognizing that it is so hard to get articles published and complete the thesis, although I had full knowledge of the Doctorate Conferral requirements when I began the PhD program. I feel regretful for pursuing a PhD, while it is a shame to quit now. I really don’t know whether to hang on or not”;

Second, personal characters (e.g., being farouche, emulative, fragile, etc.) also accounted in part for their stress. As Participant No.5 said, “I am green with envy and stressful when I see my classmates at college are well-settled down in their career while I am still struggling for a PhD, my career still being an illusion. You know, while in college, they were no match for me in any respect. I feel I am such a loser.” The feeling of being a loser was also fundamentally reflected in cases where some fragile PhD students failed to make true of their expectations; Two participants also claimed that when their peer PhD classmates published articles or made any other achievements, they could not fall in sleep, feeling depressed and stressed, and one participant even suffered from long-term insomnia.

Further, the economic plight they were in (especially when it formed a sharp contrast with the wealth of their non-student peers) and having no-sex (which is exclusive to single participants) also contributed to their stress.

## Discussion

As is revealed from the data, the triggers for PhD stress were diverse and complex. The participants interpreted or dictated their own ascription of what caused their stress, and all participants experienced at least one source of stress.

Apprehension at not completing the PhD program on time was described by all the participants as the most important factor stressing them out, which echoes the research findings by Elisabeth Pain (2017). This stress is closely related to academic policy, personal factors, and supervisor guidance. After all, to complete a thesis of 100,000 words and get two articles published in core periodicals indexed in CSSCI in 3 years is rather tough (if not totally impossible) for inexperienced students. Our survey revealed that the thesis requirement stressed them out, in that they felt there was not adequate time for them to complete a thesis to the satisfaction of their supervisors, internal and external examiners on the oral defence panel or examiners spot-checking PhD thesis for assessment, due to such reasons as lack of clear objective for enrolling on a PhD program, being engaged in other activities irrelevant to PhD study (especially those enrolled in an on-the-job PhD program), not well-prepared for the toil and hardship of embarking on a PhD (e.g., ignoring the fact that a PhD is not only intellectually difficult but it calls for discipline and stamina), receiving little or no guidance from supervisors, or negatively affected by other factors. As the time to finish their PhDs was quickly running out, they had to rush through completing their thesis; as a result, some of the rushed theses were “nothing more than a casual compilation of words or slapdash”, as remarked by a professor at Nanjing Normal University who was quite annoyed by the poor quality of a PhD thesis when spot-checking PhD theses for assessment and doubted the true value of doctors whose PhD theses were written poorly. His view was shared by scholars of other institutions of higher education (Zhao & Sha, 2016). This is true with other countries. It is said that anyone who visits the digital theses and dissertation portals on the library websites of some universities will sure see the tragicomedy called post-graduate research in Kenya, i.e., some PhD theses were rushed, so cannot be published anywhere in the world (Odhiambo, 2016). In a sense, the current 3-year PhD program exerts extra pressure on inexperienced students, resulting in negative effects on PhD quality, student satisfaction, and student mental health, with few formal obligations on those that implement it. This is not only arguably unethical, but unproductive. So, it is proposed that current program should be given more flexibility (e.g., implementing a 4- or 5-year PhD program, while the most distinguished students can graduate ahead of schedule), so as to positively support PhD students completing timely PhDs, meanwhile maintain good career and mental health outcomes for the students;

Thesis apart, publication requirement exerted more pressure on graduation. The requirement of publication of two articles in core periodicals is intended to promote the quality of doctoral education for preparing students to participate in research cultures, based on the idea that some pressure to produce cutting-edge research is necessary to motivate scholars early in their careers to focus on research advancement, and learn to balance its achievement with the other responsibilities of the professorial role. However, in a climate of increasing pressure to publish articles, i.e., when publish or perish (POP) practice is widely prevalent in research-oriented universities, this requirement results in inevitable ambivalence and resistance among doctoral supervisors and candidates about the place of publication in doctoral work (Lee & Kamler, 2008). This POP practice is described as “tyranny”, for “The academic profession has become obsessed with quantity rather than quality. [..] One brilliant article should outweigh one mediocre book” (Paglia, 1991), thus is widely criticized for decreasing the value of resulting scholarship, as scholars must spend more time scrambling to publish whatever they can get into print, rather than spending time developing significant research agendas (Decca, 2013).

In China, the publication requirement is even more unreasonable or impracticable. One reason is that the limited number of periodicals listed in CSSCI is completely out of proportion to the overwhelming number of people desperate for publication; Another reason is that the academic community is not fully immune to problems such as bias and discrimination, as is evidenced by an implicit or unspoken rule that academic periodicals simply give preference to papers submitted by professors or doctoral supervisors, or those papers funded by national or provincial projects (allegedly in order to foster the reputation of periodicals), which makes article publication by PhD students almost an illusion. Interviews revealed that, except for those students who studied hard while their supervisors were considerate or had good “guanxi” (关系, literally means “relationship” or “relation”) with the chief editors of core periodicals, PhD students could hardly have publications. After all, “guanxi” plays a significant role in personal development, for it is essentially a set of interpersonal connections that facilitate exchange of favours between people on a dyadic basis; it is not merely a relationship, but a tie through which parties exchange valued materials or favours (Bian & Ang, 1997). In a sense, publication requirement actually involves the competition of supervisor background or “guanxi”, which inevitably encourages undesirable behaviour or unfair competition. It is obvious that the policy-makers ignore the fact that publishing papers is a tricky and time-consuming, business, if not completely fatal. As a result, this requirement, being no more good to improve PhD students’ academic ability than put extra pressure on students, has become a subject of public denunciation in academia (Qu, 2017; Zhang, 2018).

Job-related stress is also related to academic policy and employment situation. From 2007 on, the number of students enrolled in a PhD has maintained at 50,000 per year, a striking increase compared with 18 PhD students in 1979 when PhD candidates in China were enrolled for the first time; Meanwhile, personal factors also count. Our interview reveals that some of the participants are the so-called “fast food generation”, i.e., their motivation for enrolling in a PhD is to find a job with a good salary, to meet the requirement for conferring academic title, or just for the sake of a PhD title, etc. They focus on their short benefits, neglecting the fact that doing a PhD is hard. As a result, increase or surplus of PhD students, interest-oriented motivation for enrolling a PhD, along with gender discrimination in employment practice, inevitably lead to poor career expectations or aspirations of most PhD students, consequently, stress or anxiety results. These findings corroborate with previous research findings (e.g., Levecque et al., 2017);

Relationship crisis is even more tricky. The supervisor–student relationship is both personal and professional, and the best of supervision should be for both supervisor and student to acknowledge each other, recognize the power differential but emphasize the support of each other (Chamberlain, 2016), resulting in the supervisor being a good program manager and lifelong friend of the PhD student. But regretfully, the supervisor-student relationship revealed by most participants is that of negative image, i.e., in the process of earning their doctorate, students are subject to the power that supervisors exercise over students’ research, progress and career. This abuse of power is either of professional nature, corroborating with other research findings of darker stories of selfishness, power and meanness, where supervisors use their students to produce the supervisor’s academic work, take all the accolades for publications and results, and belittle student’s different approaches (Wisker, 2014); or the abuse of power is of personal nature, corroborating with a case where a supervisor was alleged to have committed sexual harassment (see: http://society.people.com.cn/GB/42733/8769768.html) and a recent alleged sexual harassment scandal involving a professor (Zou, 2018), though these cases are just the tip of the iceberg. Factors such as gender, social status, and social connections or networks will influence the extent to which students get bullied; Further, non-supportive family or companion, as well as intense roommate relationship also contribute to PhD stress. These results also corroborate findings that negative family relationships can cause stress, impact mental health and even cause a mental illness to worsen (e.g., Ha & Granger, 2016; Jang, Zippay, & Park, 2012). Our data also reveal that stress is more prominent among female students, corroborating with previous research findings (e.g., Gu, Levin, & Luo, 2017; Morse & Dravo, 2007).

Further, economic situation also constitutes a trigger for stress, corroborating with previous research findings (e.g., Appel & Dahlgren, 2003; Kurtz-Costes et al., 2006; van der Haert et al., 2014).

Anyway, these findings indicate that stress or anxiety is prevalent in PhD students; so, there is a real need for PhD programs to actively support candidates completing timely PhDs. Proposal for progressive solutions includes: 1) improving supervisor training and management to ease the intense relationship between students and supervisors; 2) adopting a more flexible PhD program or reforming current PhD education policy to improve student outcomes whilst provide strong incentives for candidates to finish in a timely manner; and 3) establishing relatively complete counselling service. Participants in this study described stress as a salient experience closely related to their interpersonal relationships (with family members, class mates, supervisors, etc.), identity development, and career decision-making processes, etc. As a result, career practitioners, psychotherapists, family and supervisors are encouraged to provide counselling. After all, counselling is necessary and vital for the psychological well-being of PhD students (Anderson & Niles, 1995; Blustein, 2006).

## Conclusion

The findings depict various social, economic and academic factors for stress experienced by 10 Chinese PhD students. The qualitative nature of this study is contributory to the merger but emerging body of literature on mental health crisis in graduate education by illuminating the phenomenological experience of it among PhD students, and corroborates a number of anecdotal evidence, personal strategies or concerns revealed on social media or elsewhere. It is envisaged that the findings of this research will provide some new evidence for institutions of higher education and result in certain reform in respect to PhD education.

Recently, some influential periodicals (e.g., *Nature* and *Science*) have reported academic concerns about the psychological well-being of PhD students, arousing much concern at national and international levels. Relatedly, the first limitation of this study is that data collection at such a time may have heightened sensitivity to experiences related to stress for PhD students and influenced participants’ responses; The second limitation involves sampling. All participants, recruited from seven universities in three provinces in China, were majoring in humanities and social science. Thus, lack of diversity of various backgrounds means these findings represent an incomplete dictation of triggers for anxiety or stress, thus cannot be generalized to PhD students of other backgrounds. Further research with more diverse samples, e.g., geographic region, students of other backgrounds, etc., is needed.
